# Determinants of Infant Young Child Feeding Among Mothers of Malnourished Children in South Punjab, Pakistan: A Qualitative Study

**DOI:** 10.3389/fpubh.2022.834089

**Published:** 2022-05-19

**Authors:** Farooq Ahmed, Najma Iqbal Malik, Muhammad Shahzad, Manal Ahmad, Muhammad Shahid, Xing Lin Feng, Jing Guo

**Affiliations:** ^1^Department of Anthropology, Quaid-i-Azam University, Islamabad, Pakistan; ^2^Department of Anthropology, University of Washington, Seattle, WA, United States; ^3^Department of Psychology, University of Sargodha, Sargodha, Pakistan; ^4^Department of Anthropology, Bahauddin Zakariya University, Multan, Pakistan; ^5^Mather Hospital Northwell, New York, NY, United States; ^6^School of Insurance and Economics, University of International Business and Economics (UIBE), Beijing, China; ^7^Department of Health Policy and Management, School of Public Health, Peking University, Beijing, China

**Keywords:** feeding practices, breastfeeding, barriers, South-Punjab, Pakistan

## Abstract

Inadequate feeding is one of the most critical underlying determinants of child malnutrition. In this study, we explore infant young child feeding (IYCF) and deconstruct breastfeeding barriers in mothers of severely malnourished children in one of the most marginalized districts of Punjab province of Pakistan. Using purposive sampling, 20 lactating mothers are recruited for open-ended semi-structured interviews. Results reveal that barriers to immediate and exclusive breastfeeding include the introduction of pre-lacteal, butter, and cow or formula milk by mothers and grandmothers. Birthing difficulties and ritualizing prelacteal to transfer religion and culture cause the delay of early initiation of breastmilk. The colostrum is also discarded based on its weird physical look. Moreover, household circumstances, limited diet, extra workload, and mental stress associated with marital relationships are other significant barriers. Mothers perceive their breastmilk as thin, impotent, and of bad quality and often complain against breastmilk insufficiency due to general weakness. Furthermore, poor mothers reduce breastfeeding when the fertility burden is high, especially if a female baby is in their womb. Alternatively, outer milk is recommended but washing bottles with detergents often becomes frequent. In conclusion, immediacy, exclusivity, frequency, and duration of breastfeeding are circumscribed owing to multiple social, cultural, and economic causes. Therefore, a holistic approach combining cultural and structural causes might be more relevant for successful IYCF practices in marginalized communities of Pakistan.

## Introduction

Worldwide, 38% of infants are exclusively breastfed every year, causing infant mortality due to suboptimal breastfeeding (1). Pakistan's progress in newborn and child health and nutrition is even lower than other low-and-middle-income countries, as <20% of mothers timely and exclusively breastfeed (2). The first 1,000 days of a newborn, known as the window of opportunity, are considered the most crucial time of child growth. In line with this, the main reason behind half of global child mortality in the year 2011 was inadequate or inappropriate feeding practices (3). The World Health Organization (WHO) (4) and United Nation International Children's Emergency Fund (UNICEF) (5) recommend breastfeeding to babies for “two complete years” without the introduction of any other food in the first “five to 6 months” for an infant. After 6 months, however, a baby requires some extra “complementary diverse foods” along with continued breastfeeding for at least 2 years (6, 7). During this time, the diet of a lactating mother must therefore must be adequate, rich, and diverse enough to optimally breastfeed her baby (8). Optimal infant young child feeding (IYCF) practices do not only determine physical growth and cognitive development in childhood but also in later adulthood health outcomes (9, 10).

Biomedically, immediate and exclusive breastfeeding are highly recommended to the newborn after birth. However, several mothers ignore these recommendations owing to numerous reasons. Late and low quantity breastmilk is often initiated, especially after a cesarean or performing rituals of pre-lacteal that potentially upset the priming of the intestinal tract (11). Meanwhile, animal milk or infant formula milk is alternatively recommended by the majority of health professionals. Young, illiterate, poor, and working mothers have very few options except adopting various sub-standard feeding practices often due to overwork burden, low income, lack of awareness, and upon the suggestions of close neighbors, relatives, grandmothers, or even the biomedical community (12, 13). Some studies indicated that barriers to exclusive breastfeeding included mothers' perception of the insufficiency of breastmilk for the infant. For example, in Kenya, malnourished mothers perceived their milk as nutritionally inadequate to satisfy child hunger (14). There is a dearth of studies that highlight the determinants of IYCF adequately (15, 16). As both cultural and structural factors play a decisive role in optimal breastfeeding among poor mothers (17), only a few studies in India and Pakistan have genuinely discussed IYCF practices and their underlying causes that are embedded in the social, cultural, economic, and environmental configuration (18).

The existing qualitative literature on sociocultural and economic determinants of IYCF practices in low-income working mothers in South Punjab is sparse (19). In the past, however, few studies in Pakistani Punjab have highlighted a deeper understanding of social, economic, and structural determinants like poverty, illiteracy, lower status, and inadequate care of women (20, 21). The immediacy, exclusivity frequency, and duration of breastfeeding behaviors depend upon the complex socio-cultural and economic circumstances of mothers. It seems unjustified if IYCF is understood without a broader context. Therefore, this study aims to reveal deeper sociocultural factors that are linked with sub-optimal feeding practices at community levels in Rajanpur, one of the least developed districts. This study explores the significant causes behind suboptimal IYCF beliefs practices. What are the rationales behind delaying the early breastmilk introduction and the action of mothers discarding their first milk from breasts? What are the worries of novice mothers and how far is social support essential for early breastmilk initiation soon after delivery? How important is household income, husband's employment, peace at home, and time for the care of a baby? These are the questions the present study principally attempts to respond to in this article.

## Materials and Methods

### Research Design

The qualitative data used in this study was collected during ethnographic and intensive fieldwork in the Rajanpur district. District Rajanpur was purposefully selected because it has historically shown the highest prevalence of maternal-child malnutrition rates (~50% maternal anemia; <50% stunting and underweight children) compared with other districts of southern Punjab Pakistan (15). Along with the highest poverty rates (60%), food insecurity, severe water insecurity, and lowest literacy levels make people more vulnerable in remote rural areas, especially females and pregnant and lactating mothers (19). Overall, the district shows a depiction of south Punjab as having insufficient health and education facilities and an increased poverty rate (22). The literacy rate is significantly low (29%) for males and only 11% for females. The prevalence of contraception is also extremely low in the district (23). In the absence of qualitative evidence related to IYCF barriers among mothers of severely malnourished children in Pakistan, the current study was based on a qualitative descriptive research design that employed in-depth interviews. This research design permitted us to sample and access the most affected mothers to capture the social, economic, and cultural determinants behind suboptimal infant and young child feeding. The study restricted in-depth interviewing to lactating and pregnant mothers with children suffering from severe malnourishment. To capture feeding practices, the inclusion criteria for recruiting mothers necessitated them to have been lactating for not more than 3 years.

### Interview Guide Development

We developed a semi-structured interview guide (see Supplementary Material) for interviewing mothers after an extensive review of the literature using keywords related to the socio-cultural determinants of IYCF. Also, we pilot tested the interview guide with a total of 6 mothers prior to data collection. Furthermore, we also updated the interview guide during fieldwork from time to time whenever more information about the community's feeding practices was revealed. Interviews were open-ended with a flexible format, so multiple issues were discussed during in-depth semi-structured, and open-ended interviews, which ranged from 1 to 2 h. Local language (Seraiki) was used to write down the notes to maintain a flow of conversation between the interviewer and interviewees.

### Data Collection

The nutrition assistants and lady health workers (LHWs) in the Community Management of Acute Malnutrition (CMAM) program identified mothers of malnourished children. Nearly 30 parents and their immediate families (living under one roof) were informed about the nature of the study to seek their consent and willingness to participate in the study. However, 20 selected mothers volunterily agreed to participate in the study, and mothers gave their oral consent during pre-interview meetings. For data collection, purposeful sampling was preferred over random sampling. Purposive sampling helped to gather specific information on different issues. Parents, especially mothers, were interviewed and inquired, and an interview guide was used. Probing was excessively used, and dialogical communication was set up to gather the qualitative type of data. Data were collected from February 2017 to May 2017. We conducted semi-structured in-depth interviews with 20 mothers. Mothers were first introduced at the local health facility and then interviewed in their own houses. Their places were chosen to provide them with a safe and comfortable environment so that mothers could easily share their beliefs and practices about feeding. Well aware of the local language and culture, all these interviews were conducted face-to-face in the local language by the two authors (FA and MA). Seeing the cultural sensitivity and comfortability of the respondents of the study, no audio recording was done for the interviews, and that is a limitation to the data analysis. Most of the respondents were illiterate or had attended school for 1–2 years. The majority of mothers belonged to lower socioeconomic status (~$100/month) and were either agricultural laborers or domestic household servants (Table 1).

**Table 1:** Respondents' sociodemographic information.

**Respondent**	**Age**	**Literacy**	**Children**	**Occupation**	**Household income (PKR)**
Mother 1	25	Primary	4	Domestic labor	10,000
Mother 2	30	No	1	Agricultural labor	8,000
Mother 3	30	No	3	Domestic housewife	7,000
Mother 4	35	No	6	Domestic housewife	11,000
Mother 5	24	No	3	Laborer	8,000
Mother 6	33	No	4	Agricultural labor	15,000
Mother 7	28	No	2	Domestic HH servant	13,000
Mother 8	16	No	3	Dailywage worker	6,000
Mother 9	34	Primary	7	Agricultural work	8,000
Mother 10	29	No	5	Agricultural labor	10,000
Mother 11	40	No	8	Agricultural labor	9,000
Mother 12	40	No	6	Agricultural work	8,000
Mother 13	38	No	5	Manual Laborer	6,000
Mother 14	30	No	7	Domestic HH servant	7,000
Mother 15	36	High	2	Housewife	40,000
Mother 16	35	Middle	3	Teacher	30,000
Mother 17	25	No	3	Domestic work	14,000
Mother 18	17	No	6	Domestic work	10,000
Mother 19	31	No	4	Domestic work	9,000
Mother 20	24	No	7	Domestic HH Servant	16,000

### Data Analysis

**Figure 1:**
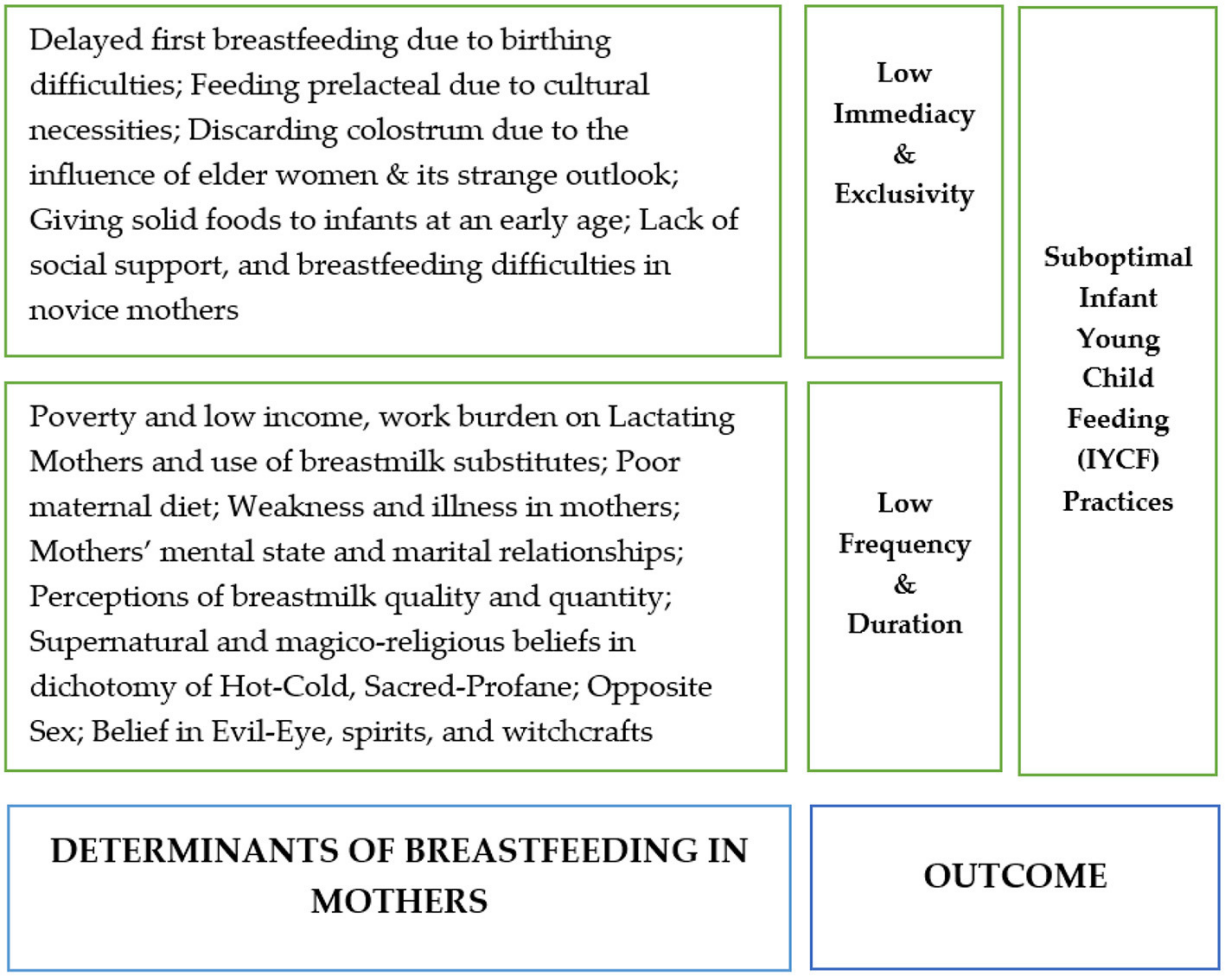
Major barriers behind optimal infant young child feeding (IYCF) practices among mothers of severe acute malnutrition (SAM) children.

Interviews and field notes were instantaneously translated verbatim into the English language. After an intensive review of all translations and field notes by the two authors (FA, MA), the qualitative data were analyzed through a manual thematic analysis approach. Using inductive and deductive methods, all raw data was perused and thoroughly overviewed. Then, sentences and texts were labeled into different codes. These codes permitted us to find out the common meanings and group similar ones to create broader categories. After reaching consensus, the conceptual categories were described, and a reverse check was run examining categories and corresponding significant statements in the data. Through cross-verification of the narratives, the vagueness, discrepancies, and similar codes were removed. In the end, we analyzed codes and categories and accumulated different sub-themes and themes that affected immediacy, exclusivity, frequency, and duration of breastfeeding and ultimately resulted in sub-optimal IYCF practices in mothers of children with severe acute malnutrition (SAM; Figure 1).

### Ethical Consideration

Ethical approval for this study was obtained from the Department of Anthropology and the advanced study and research board (ASRB) of Quaid-e-Azam University Islamabad in its 307th meeting held on 20 October 2016 (Number: QAU-ASRB-2016-307; Date of Approval: 20-10-2016). The board resolved to approve the recommendations of the Dean of the Faculty of Social Sciences to approve the current ethnographic research in the Department of Anthropology. All respondents were first informed about the nature of the study and then requested to participate with free will. We obtained the oral consent only because most of the study respondents were illiterate. Furthermore, requesting them to formally put signatures on papers might have caused confusion, doubt, and evasion. Keeping in view the ethics, we ensured the privacy, anonymity, and confidentiality of participants.

## Results

### Factors Affecting Immediacy and Exclusivity of Breastfeeding

The early initiation of breastmilk and exclusive breastfeeding were influenced due to several reasons.

#### Delayed First Breastfeeding Due to Birthing Difficulties

Although normal birthing was frequent, few respondents (*n* = 3) also reported cesarean section (CS). Irrespective of the mode of delivery, most mothers informed that it was a difficult time and to breastfeed immediately after birth was delayed 3 to 4 days due to painful experiences of deliveries. Therefore, infants were meanwhile fed on bottled milk. Interestingly, few mothers uncovered inappropriate counseling by the untrained health staff, which affected breastfeeding.

“I breastfed late because the delivery was stressful and painful for me…. therefore I even didn't like to look at my baby after delivery.” (M15)

“I breastfed my baby after 4 days of delivery as it was tough to feed the baby……in the meantime the baby was given infant formula milk as the first feed.” (M18)

“Tummy gets bigger if you drink plenty of water after delivery.... so I stopped drinking water and milk in my breasts couldn't be produced, therefore my baby was fed very late and meanwhile was fed on bottled milk.” (M11)

#### Feeding Prelacteal Due to Cultural Necessities

Newborn babies were given energetic, warm, sweet things, and sometimes herbs as prelacteal (*Ghutti*). This tradition was socially conventional, traditionally meaningful, religiously supported, and culturally perceived as medicinally significant to welcome the newborn baby. There were different cultural conceptions associated with prelacteal feed, such as that it cleans the birthing filth, purifies the child's body, and transfers the nobility. However, feeding prelacteal was not always hygienic and exposed neonates to infections. Breastfeeding was believed to be a blessed action, and for that reason, natives initiated it only after profane waters from the newborn's body were discharged through prelacteal. A mother of three children reported that:

“We always give jaggery or honey after birth. Its essence is warm and cleans dirty fluids from newborn body and neonate ought not to be given cold things soon after delivery since the newborn has come out of mom's warm tummy.” (M3)

#### Discarding Colostrum Due to Its Strange Outlook

Mothers discharged colostrum from their breasts by pressing and squeezing either manually or with the help of a machine or tool that is available in the market. Birth attendants also helped mothers to pour this thick and sticky material out of their swollen breasts. Some mothers even paid little mount to traditional midwives who emptied their breasts, which indicates how colostrum wasting has become a trend. The majority of mothers perceived first milk as disadvantageous, and only a few females perceived that it should not be wasted because it is “energetic.” Most mothers believed that colostrum was unsuitable for the newborn's stomach because it was thick, sticky, and yellow. These reasons were compelling, which uncovered the participant's beliefs and conceptions of mothers based on the physical appearances of its matter, smell, viscosity, stickiness, and gumminess. Mothers supposed that there was gumminess (*cheerhon*) in its configuration. It was painful and difficult to digest condensed matter and caused swelling in the mother's breasts some months before delivering a baby. One mother explained this issue as:

“They do not use colostrum as old women of our area believe it causes “aara” (difficult to swallow) due to gluiness and thickness, swelling in the intestines, and constipation in infants.” (M17)

Another mother similarly informed:

“We avoid this because it sticks to the food pipe (esophagus) of the child and causes infection; senior women of our side advise this [colostrum] is sticky and causes swelling in intestines and constipation.” (M5)

For some mothers, the primary reason for the outage of first milk was the bad smell in colostrum because of its prolonged stay inside the breast:

“The smell of old milk is of acidic sour (sandhan) because of its months-long stay inside the mother's breast, let it be ousted so that regular milk could start pouring out from the nipples. The standard liquid does not flow unless this decayed milk oozes out by squeezing and pumping the mother's breasts.” (M15)

Mothers perceived that the essence of the first thick milk was hot and risky for an infant. One mother argued that they discarded it because it was warm and spilled milk (*phaita*).

“We don't give because it is hot, and it makes the baby ill…..the baby should not be given breastmilk in summer because it gets warm when we work during hot days of summer; therefore, the baby gets sick.” (M3)

“It is expired and old material collected due to longer a stay. Also, its color is yellow, and it looks like infection and pus; it gives the feeling of abomination, and it can't pass through the baby's throat.” (M9)

One mother argued that since yellow and red are in contrast, they should not be mixed. Its color has now turned yellow, and it is not good for blood [of red color]. It can spoil a baby's blood and may also cause diarrhea if given in big quantities. One mother justified her belief as:

“This old milk is not good for a newborn because it is stored in the mother's breasts for several months. It expires, turns yellow, and becomes toxic so it must be immediately wasted. The breasts swell because of deadly substances. It looks like an infection and is unsafe to keep in the breasts for a long time.” (M20)

“First milk is thick, heavy, and causes constipation, and stops first excreta (daasa). Also, it is adhesive and sticky to the fingers just like oil, so it is much heavier to the intestines of a little child.” (M8)

#### Discarding Colostrum Due to the Influence of Elder Women

Novice mothers have trust in the experiences of their mothers and grandmothers, who easily convince young and newlywed women to follow their instructions.

“I had a strong faith in my mother's experience who advised me to waste early milk. My baby was born underweight; I was so worried about her rare condition. So, I wasted my yellow, thick, glossy milk on a pumping instrument that was already present in my grandmother's house. She used to lend it to everyone in such a situation.” (M15)

The practice of excreting colostrum is common and is being followed and transferred from generation to generation, from grandmothers to mothers. Of mothers who wasted colostrum did it on the advice of grandmother or mother or mother-in-law, as explained by a mother:

“I lost my colostrum on the suggestion and guidance of my mother; I was unable to breastfeed my infant due to unhealed stitches after C-section. My breasts were exceptionally pulled up; I used to feel much pain in my breasts as well as underarms. My mother helped me in massaging my breast with sesame oil and water, then I excreted first thick milk through a hand pump, it was almost half cup of yellow, thick and glue and gel type of milk; then I added it into the mug of water which became thick and yellow.” (M7)

#### Lack of Social Support and Breastfeeding Difficulties in Novice Mothers

Novice mothers need guidance during pregnancy and lactation. Most of the mothers often depend on prevailing customs, therefore, the social environment plays a positive role if provided by the family, hospital staff, nurses, husbands, mothers, and grandmothers. One mother (M9) explained:

“In pain, I was unable to breastfeed, my breasts were swelling because of thick milk in them, but nobody guided me to give early milk to the baby; in the first 3 days, my baby could not drink breastmilk even though I was discharged from the hospital.”

“Transitional milk did not come after the third day of delivery due to tensions but my family advised me to nurse your baby because it protects a woman from diseases; therefore, I tried again and again, and ultimately my breasts were engorged with milk.” (M16)

Contrarily, one mother revealed that she kept on breastfeeding her baby improperly for a long time until one of her relatives noticed her breastfeeding the wrong way and taught her the correct method.

“Worried, she said your baby is not sucking your milk, do you know that, is this the sucking sound? You don't know how to feed a baby. If you permit me, I can help you breastfeed properly. I gave her permission. She then cupped my breast from the bottom and entered the nipple in the baby's mouth, and the baby started sucking milk from the breast, and plenty of milk went into the baby's stomach through the mouth this way. I soon realized that I had wrongly breastfed since “Day 1.” I lamented how, due to my lack of experience, my baby remained hungry as I could not correctly feed my baby due to ignorance.” (M12)

#### Giving Solid Foods to Infants at an Early Age

Findings showed that mothers introduced different foods, such as biscuits, tea, water, fresh butter, rice, fruit, and yogurt, even at the age of three, four, and 5 months. Many mothers believed that some water drops were vital because of infants' dried lips. It was not unusual for mothers to give their infants water, rice and butter, and biscuits during this time. However, some mothers introduced foods other than these, such as sabodana and egg. There is a local practice of giving a baby sweet homemade butter (*Junj*) at the age of 2 months. Mother and child are forbidden to drink water after eating butter as it is perceived to cause respiratory problems, chest wheezing, and nose flu. Some mothers add things into butter to make it more delicious so that the baby can eat as much as he likes. Grandmothers and mothers-in-law advise young mothers to introduce these things. One mother revealed:

“From a very early age, I use butter and add crystallized salt and sugar (noushadar and misri) that makes it thin and delicious. First, the baby weeps and does not eat it well and it can come out of the baby's nose. As butter is soft, so if you press the baby's nostrils it goes into the belly.” (M16)

It was reported that on the fourth month of birth, economically better-off people gave eggs, yogurt, and fruit, while poor parents offered only buffalo, cow, or goat milk along with potatoes.

### Factors Influencing Frequency and Duration of Breastfeeding

The total period of breastfeeding (duration) and the number of times (frequency) in a day a baby is breastfed has vital importance for a growing child. Even low birthweight babies reportedly recovered as breastmilk frequency increased. One mother reported:

“My first baby who was born weak started to grow up rapidly as soon as the frequency of breastfeeding was increased…..she used to be breastfed every 20–30 min. In very little time, she gained weight.” (M10)

“When a baby inserts foot's toe in her or his mouth, the baby is believed to have drunk 40-kg of breastmilk.” It implies growth depends on a large quantity of breastmilk.” (M13)

The frequency and duration of breastfeeding are influenced by multiple factors.

#### Low Income, Work Burden, and Use of Substitutes

Mothers stated high work burden and low income influenced breastfeeding habits that led to the use of formula milk. Working mothers complained that the frequency of breastfeeding was interrupted, and they could not satisfy their children because of their work burden. One mother (M7) stated:

“My second child is a daughter who is one and a half years old who often gets sick. I have breastfed her for just 8 months and stopped breastfeeding because I had to go to work. When I used to return from work, I was too exhausted to feed my baby.”

One mother stated:

“Bigger son drank my milk for 2 years. The others just drank for 2 months because I had to leave my house for work as a household domestic in the homes of better-off people to earn some money; therefore, I used to give bottles to my babies, so they gradually left my breast.” (M1)

Sometimes, babies of working mothers were taken care of by grandmother, siblings, or other close family members who prepared formula milk in their absence and gave feeders that were improperly washed. One mother (M20) expressed:

“As I go to work daily, and I had to leave my children in the custody of the grandmother. My son gets sick very often because of bottled milk, most of the time, due to an un-cleaned feeder by his grandmother in my absence. The immunity of my son is deficient as is he often sick.” (M2)

Another woman stated:

My children eat sand or mud from the wall and floor. Ideally, a mother needs to look after her children but where I work as a domestic household servant do not allow to bring my children.” (M14)

#### Psychological Stress and Marital Relationship Influence Breastfeeding

Good income and mental peace at home were perceived by many mothers as a precondition for good infant young child care and feeding.

“Both mother and father are necessary for children's care. I am a single mother; their father has left us. How can a mother properly take care and breastfeed if there is no peace at home? My breastmilk has become dry due to this everyday stress and violence.” (14).

“After marriage, the most important thing for a mother is living in-laws. The mother should be happy because a healthy child requires a happy mother.” (M9)

Many mothers reported that they could not breastfeed their babies due to repeated pregnancies and a high fertility burden. Breastfeeding the female gender was reported to be much shorter than male children because of the social construct that “the daughter is the property of the husband after marriage, but the son is our child.”

#### Maternal Diet Influences Breastfeeding Duration

Low-income mothers illustrated how maternal diet in the household is associated with the duration of breastfeeding.

“I have no milk…..I breastfed for just 2 months, and then my milk dried up because I was already much weak, and my diet wasn't very energetic. We can only avail of wheat, potato, and onion. I have often asked my husband to bring some meat and fruit.” (M2)

Mothers need diverse food, frequent diets, time, and care to optimally breastfeed their infants. According to mothers, low household income potentially reduces the frequency of breastfeeding, and if mothers breastfeed more than their capacity, it makes them malnourished. The child grows after challenging the activity of breastfeeding. One mother (M4) expressed:

“When I get up in the morning, my face looks healthy……I start breastfeeding after breakfast and continue till evening, at several intervals…..in the evening, my face looks dried up, especially when the diet is low I feel very exhausted….breastfeeding is not a joke it takes enough energy from the lactating mother. Of course, a poor and malnourished mother can't satisfy the baby with her breastmilk.”

Mothers feel exhausted without adequate drinking and eating. Therefore, regular diet maintenance, especially during illness, is strongly required. For poor mothers, breastfeeding becomes much more challenging.

#### Perceptions of Low Quality and Quantity of Breastmilk and Illness in Mothers

Some mothers perceived that the baby was weeping due to breastmilk's bad quality and insufficient quantity. Therefore, company milk was a suitable option. One mother (M6) stated:

“My milk is bad, thin, child cry with this and kicks me; why should I prefer breastmilk, which is no thicker than formula milk, it looks like muddy water (mela pani), which has no power to make my baby fat? Why should I not use formula milk, which is thicker, tasty, and looks like pure milk?”

Illness makes mothers reluctant to breastfeed their babies, as in their opinion, milk becomes polluted and causes morbidity among babies. Only a few mothers believed that the child must be given breastmilk during sickness, and women who do not give in sickness are wrong. One mother said, “it is a kind of great sin and doing like the shirk (equating God with something).” On the other hand, many lactating mothers quit breastfeeding as the breastmilk of a pregnant mother may cause illness. Some mothers prefer their contaminated and poisonous milk tested by a so-called experiment.

“We collect a small sample of liquid from an ill mother in a spoon or small cup and insert an ant or honey bee into this. If the ant dies inside milk, they believe that it was due to the poisonous milk of the sick mother and therefore had become non-drinkable, and if an ant or bee remains alive, the milk is perceived as healthy and drinkable.” (M11)

It is also believed that milk becomes stale and bitter after taking bitter pills during illness, and the habit of breastfeeding is gradually reduced.

#### Perceptions in the Supernatural Beliefs (Sacred-Profane and Evil-Eye)

Some mothers believe that breastfeeding a baby with the same sex as the one she is pregnant with [one drinking breastmilk and other inside the belly] is not dangerous, but breastfeeding a baby of the opposite sex of the unborn might be harmful to the baby being breastfed. In their opinion breastmilk is polluted if sexes differ. One mother expressed:

“If a mother is pregnant with a male sex child and she breastfeeds a boy child, it doesn't harm suckling baby.” (M7)

It is supposed that breastfeeding can be interrupted due to jealousy and the evil intentions of others by spirits. Some mothers believed to “never breastfeed along with wet hair unless lactating mom tightly holds hair-curl in the mouth.” Also, it was perceived that a mother should not make a cat frightened especially in the first 40 days of lactation. Otherwise, spirits may transmit from animal to human and harm breastfeeding. Furthermore, breastfeeding should not be preferred soon after an inauspicious event:

“My mother stopped me from breastfeeding and asked first to wash my breasts because I was coming from an ill-omened ceremony, which might bring misfortunes for a small baby. I obeyed my mother and acted upon her advice, as it seemed appropriate to me” (13).

Breastfeeding may be affected by intercourse if it is done without a gap and after cleaning and intercourse. It is considered taboo in Islamic society. An act of breastfeeding ought to be restricted right after intercourse because it is thought that an infant child may drink milk from the profane body of the mother. Since milk is conceived as sacred and sex as lewd, a due gap is obligatory. Hence, bathing might fill this gap prior to breastfeeding the baby, otherwise, consequences such as deviance in later life are due. One woman stated:

“If a wife sleeps with her husband she cannot breastfeed her baby without taking a bath, as the child eats illegitimate (haram) food, becomes an illegitimate baby (harami)...pregnant women should not continue intercourse after the 6 month, otherwise, the child might become a criminal (Jurmi), and tend to commit illegal acts and moral crimes in the future.” (M18)

## Discussion

This study analyzed mothers' perceptions and practices regarding immediacy, exclusivity, frequency, and duration of breastfeeding in mothers of SAM Children. Results revealed that immediacy, exclusivity, frequency, and duration of breastfeeding were restricted due to multiple cultural, religious, and economic factors. Mothers indicated that they could not get proper counseling at the time of delivery. Hence, breastfeeding immediacy was hindered due to the miscommunication and inappropriate counseling skills of health staff. Previous studies have also testified of the inappropriate counseling skills during pregnancy. In addition, the immediate post-delivery period at the maternity homes and hospitals (24, 25) remains a serious public health concern. Inappropriate counseling is often linked to various types of misconceptions and social disbeliefs that prevail ubiquitously across sectors. A combined social effort or movement is a crucial in this regard.

According to the majority of lactating women, early milk should be discarded because the color is yellow, it is difficult to swallow, and the smell is stagnant. Breastfeeding early thick, yellow, sticky, and smelly colostrum is often painful and challenging, especially for young mothers. However, only a few women perceived it as vital for children's growth. The ritual of practicing prelacteal is followed that delays the early initiation of breastfeeding. Through this ritualistic practice, a transfer of religion and culture occurs. In the meantime, some formula milk is recommended, often along with breastmilk because mothers perceive that their milk is insufficient to satisfy the infant. A study from South Punjab shows that gender norms, illiteracy, the influence of family elders, and the medical community, including traditional birth attendants were found as most influential (26). Soon after some months, other foods like butter, egg, and water is started. The use of pre-lacteal as a warm feed to excrete dirt from the newborn's body and prevent infections has medicinal importance in the locals' minds. The personality construction by transferring pious qualities is also significant. In the majority of the complicated SAM cases, cow milk induced allergic reactions and severe diarrhea. Exclusive breastfeeding was not followed, and water, tea, biscuits, butter, cow milk, and loaf were used. Early introduction of foods reduced breastfeeding frequency. In several cases, cow milk caused dysentery and complicated severe acute malnutrition in infants.

The colostrum was wasted due to various reasons: delivery was horrific and painful; early milk caused constipation; it stopped the first excreta; it was difficult to swallow due to gluiness and thickness; and it caused swelling in the intestines. Mothers wasted colostrum as a preventive strategy because it was considered too difficult to pass through the baby's narrow throat. According to many women, colostrum was smelly, thick, gluey, sticky, yellow, spoiled, poisonous, expired, burned, warm, and was the result of some infection. Therefore, it was a waste. They interpreted this phenomenon through a local cultural lens (27). Spilled milk can cause illness. Gluiness, stickiness, thickness, pale yellowish color, and pain to excrete this matter make this stale milk to be disapproved and rejected. In contrast, the introduction to alternatives, such as formula milk, is considered safe because it has been prescribed by the respectable scientific community (28). Few novice mothers keep on breastfeeding in improper ways. However, some mothers who could get family support succeed to optimally breastfeed their babies. Someone's help (for example nurses, relatives, and mothers) is required to discard early painful thick milk from breasts.

An ant or a bee is inserted into mothers' milk to testify its toxicity and poison. The legitimacy of such tests is gained from reasons that include work burden on the mother, low maternal diet during lactation, illiteracy, and high fertility. If the mother is ill, breastfeeding is stopped. Some mothers quit breastfeeding during illness, believing that “polluted breastmilk” might harm the suckling baby, that milk is insufficient and poisonous, and that the use of powdered milk has links to the workload on mothers. Some mothers even further tested their breastmilk to justify their constructs (29). This research investigated breastfeeding behaviors in mothers, the barriers they faced in the smooth practice of breastfeeding, excuses for a late start, alternatives to breastmilk, and their reasons for breastfeeding cessation. These findings are in line with other studies, which state that women face difficulties in breastfeeding, perceive that milk supply is inadequate, and that the infant was not satiated (30). In Lebanon, women perceived that their breastmilk was less “energetic” and injurious to the baby (31). The mothers in other contexts similarly perceived insufficient milk syndrome (IMS), in which the child was still hungry as breasts were empty. Hence, breastmilk was perceived to be insufficient to complete the child's requirements. Meanwhile, formula was prepared until breasts were filled and again became hard with milk (32–34). As Farmer (35) observed, illnesses speak louder than words, and etiologies (primarily biologic but are culturally sanctioned causes of illness) and somatization of distress must be a form of protest by women.

The limited diet of poor females and hidden hunger also influenced the production of breastmilk. Stress and lack of peace at home is believed to dry breastmilk (36, 37), especially of household domestic female servants. Good relations with the husband and a peaceful environment in the household were encouraging for breastfeeding. Mothers busy at work or worried due to economic issues and were unable to appropriately respond to the lactational need of infants and young children. A good maternal diet can only guarantee proper breastfeeding for babies. The biomedical community often gives mothers a bulk of instruction about exclusive breastfeeding without recognition of the constraints. Encouraging exclusive breastfeeding can also tend toward deprioritizing a mother's health when she is malnourished (34).

Domestic servants and agricultural laborers were reportedly affected because of their low socio-economic status. Some qualitative studies found that the decision to quit exclusive breastfeeding was associated with paid work outside the home (38). If mothers are to prepare for the optimal feeding practices, adequate food, quality education, family planning, access to healthcare, time for care, and happy life in the in-laws are compulsory. Mother's poor health, low variety in food, and low energy intake motivate several lactating mothers to find alternatives to breasts, such as bottles, butter, early introduction to solid foods, and premature weaning practices, which become the cause of sub-optimal feeding, protein deficit, low immunity, and infection.

During illness, mothers preferred bottle-feeding because, in their opinion, they may transfer disease to the baby. The explanatory models among the community were simplistic in analyzing linguistic realities based on binary opposite constructs, contradictory laws, spiritual etiology of diseases, and nobility. The mothers believed that breastfeeding after intercourse may make a child sick. The mothers quit breastfeeding during illness. They constructed that polluted breastmilk makes a baby sick. Supernatural means of treating ailments through amulets, magico-religious, covenant, and spiritual healing methods were used. Dichotomous constructions of sweet-sour, hot-cold, and sacred-profane ultimately influenced their care, cure, and feeding practices (26, 39, 40). Mothers stopped breastfeeding after intercourse as breastmilk is a holy activity, while sex and semen is profane. Therefore, both must be separated. Similar dichotomy and separation in other ethnographies of African context showed intercourse during breastfeeding is supposed to cause kwashiorkor and malnutrition (41, 42).

The finding showed mothers wasted colostrum as it was considered hot. Mothers may have perceived this by making an analogy [of hot-cold] that milk of a buffalo or cow becomes spilled in high temperature in hot weather. The hot-cold belief system in human biologies, such as hypothermia, heatstroke, and fevers, resembles the coding of hot and cold in foods (39). In Guatemala, breastmilk is supposed to cause diarrheal diseases in the lactating child if the mother is sick either due to indigestion or evil eye when the milk becomes very cold or hot, or when hot-cold balance is altered (40). Hence, after birth, specific diet, activity, and personal care are advised. Lactation is perceived as a warm state, while postpartum is cold. Therefore, lactating women should avoid hot foods. Also, mother milk may be affected by anger or fright. Thus, treatments vary as per supposed causes, bringing changes in diet, remedies, and even complete weaning. Scheper-Hughes argued that there was always a social history of cultural practices.

Some mothers sorrowfully complained that on birth occasions, the medical community did not guide them for proper breastfeeding but recommended breastmilk substitutes, overlooking the adverse consequences of powdered milk and hygiene in bottle maintenance. Strikingly, many mothers washed bottles with “detergents” instead of dish wash bars or “washed bottles after boiling,” thereby causing severe implications for infants. Many of the mothers did not know how to properly wash bottles as they first boiled bottles and washed them with detergents afterward. In their opinion, detergent was more effective than merely washing with a soap bar. Washing bottles after boiling was useless. Mothers were advised to wash bottles, but the proper way of washing was confusing for illiterate mothers. Some mothers revealed that they used detergent to wash the baby's feeder. There were higher chances that chemicals that might have been harmful to the tiny stomach are retained inside the bottle because of its narrow neck even after washing. Mothers should be made aware of the negative consequences of formula milk feeding through community mobilization. Parents can also play the role of counselors to their children, and mothers must be informed about the best practices and curtailing difficulties in optimal IYCF practices (43). There is evidence that shows how formula milk production has constructed the practices of breastfeeding around the globe (44–46).

Mothers would lactate when alternatives and substitutes to breastmilk are readily available in the market, especially when the medical community encourages and prescribes it to parents soon after delivering babies. The gradual reduction in the frequency of breastfeeding might develop an adaptation to formula milk. The obvious result was an early weaning, but mothers blamed their milk as being the cause of a child's outcry, considered their milk inferior to formula milk, and opined that it is thin, without vitality, and power. Other studies showed similarities in South Asian countries such as Bangladesh and Pakistan (47).

As our findings show, the duration of breastfeeding a male child was longer than that of a female child. The local cultural construct, “daughter is their property, but the son is ours,” showed women's subordination in a patriarchal society. The cultural choice theory has influenced breastfeeding behaviors (48). Women's health and reproduction are not deemed fit for open and wider discussion in the public and in the syllabus. In less developed remote, rural, and tribal areas, educational activities are fewer. In this situation, engaging LHWs seems to be a solution. However, in remote health units, allocated seats for female LHWs are vacant. Half of the population in Pakistan from the poorest, rural, and remote areas are without LHWs' actual cover owing to several kinds of structural failures and administrative incompetence (49, 50).

Also, in some areas, these LHWs are not allowed to enter and make contact with household women because some males perceive that they advocate for contraception and abortion and have a doubtful moral character. Moreover, they are considered agents and representatives of the Western medical system and practice. It is here that this lack of trust restricts cultural approval. Biomedical knowledge is rejected because it is believed to be harmful not only for women's bodies but also against religious and cultural values. Behavior Change Communication (BCC) for IYCF remains less effective because it has links in sociocultural domains. The success of programs and policies about IYCF depends on a deep understanding of these social conditions, constructs, and realities. Evidence (51) shows that the policy environment of IYCF in Pakistan lacks ownership, sustainability (as only UNICEF and WHO support this), multi-sectoral collaboration, and effective advocacy or BCC. As IYCF is not the responsibility of the health sector only, other sectors especially education, population, and women welfare must come forward to contribute to this agenda along with structural reforms.

## Conclusions

This study analyzes that breastfeeding barriers and facilitators among mothers of malnourished children involve multiple social, cultural, economic, and religious determinants. Cultural constructs and religious beliefs impacted the immediacy and exclusivity of breastfeeding. Lack of awareness and family elders play a negative role in discarding colostrum. Moreover, low dietary diversity in households, work burden on poor mothers, and doctors' prescription of formula milk negatively affect optimal feeding behaviors among poor households. The study suggests that poverty, high fertility, and illiteracy make a web in which a lactating mother deprioritizes breastfeeding by presenting excuses such as the low quantity and quality of her milk. Blaming mothers and grandmothers for sub-optimal feeding only is not enough, and there is a broader context that determines breastfeeding behavior among mothers. At the same time reduction in poverty, illiteracy, and gender inequality is imperative. Although sociocultural constructs are strong barriers against optimal IYCF practices, optimal breastfeeding practices need a comprehensive analysis of the social determinants of IYCF in resource-poor settings instead of relying upon behavioral change approaches only.

### Limitation and Strength of Study

The distinguishing feature of this study is that it describes determinants of IYCF among mothers with malnourished children. It covers multiple social, cultural, economic, and political determinants, which hampers the optimization of IYCF in marginalized communities of Punjab in Pakistan. This study filled the gap in qualitative research and contributed to the field of public health, nutrition education, and medical anthropology. However, this study is inadequate, as no audio recording was done for the interviews because of the respondents' cultural sensitivity and comfortability.

## Data Availability Statement

The raw data supporting the conclusions of this article will be made available by the authors, without undue reservation.

## Ethics Statement

The studies involving human participants were reviewed and approved by Department of Anthropology and the Advanced Study and Research Board (ASRB) of Quaid-e-Azam University Islamabad in its 307th meeting held on October 20, 2016 (Number: QAU-ASRB-2016-307; Date of Approval: 20-10-2016). The patients/participants provided their written informed consent to participate in this study.

## Author Contributions

FA designed the study and conceived the manuscript. FA, MShahz, and MA collected and analyzed data and drafted the manuscript. NM, MShahi, XF, and JG contributed substantially in results interpretations, reviewing, editing, revising, and critically commenting on the manuscript. All authors were involved in writing the manuscript and approval of its final version.

## Conflict of Interest

The authors declare that the research was conducted in the absence of any commercial or financial relationships that could be construed as a potential conflict of interest.

## Publisher's Note

All claims expressed in this article are solely those of the authors and do not necessarily represent those of their affiliated organizations, or those of the publisher, the editors and the reviewers. Any product that may be evaluated in this article, or claim that may be made by its manufacturer, is not guaranteed or endorsed by the publisher.
