# Supplementary material for: Determinants of Infant Young Child Feeding Among Mothers of Malnourished Children in South Punjab, Pakistan: A Qualitative Study
Source: Front Public Health. 2022 May 19;10:834089. doi: 10.3389/fpubh.2022.834089 (PMC9160796; doi:10.3389/fpubh.2022.834089)
Supplement: Supplementary file 1 [file Table_1.DOCX]

Supplementary Material

# Supplementary Data

**Semistructured Interview Guide**

**Study: Determinants of Infant Young Child Feeding among Mothers of Malnourished Children in South Punjab, Pakistan: A Qualitative Study**

**Under Research Project:** Sociocultural Construction of Child and Mother Malnutrition in Rajanpur (No. QAU-ASRB-2016-307; Date of approval 20-10-2016).

**Sociodemographic Characteristics of Informant**

Age___________________ Gender___________________

Occupation________________ Literacy level ___________________

Household Income__________ Area/District___________________

Date___________________

Time on interview began__________ Time when interview ended__________

Name of the interviewer___________ Name of facilitator___________

**Introduction**

**Today we want to discuss some issues related to significant causes behind suboptimal IYCF practices in young children. This study explores the social, economic, religious, and cultural determinants of Child Feeding and Breastfeeding. These are the questions the present study principally attempts to respond to in this article.**

**Questionnaire**

What do you use as pre-lacteal? Honey, jaggery, raw milk, cow milk, etc. [Probe]

Do you use early milk (colostrum)? Why yes, and why not? [Probe]

What is used in the first six months, water, butter, biscuits?

[Probe other factors and ask why]

Do you think vaccination is important? Did you vaccinate your children? Which mostly [Probe]

What kind of water and milk is given to the child? Surface water, pond water, boring water etc. [Probe]

Breastfeeding duration: how many months were children breastfed in total by the mother? [Probe]

Did you breastfeed during pregnancy? [Probe]

What are your perceptions of breastmilk quality and quantity? [Probe]

Do you think children should be breastfed during illness and after sex? If not, why? [Probe]

What is the role of religion and faith in breastmilk feeding?

Religious beliefs in the hot-cold and sacred-profane dichotomy evil-eye, and witchcraft, [Probe]

Who cares about the child in the mother’s absence? [Probe]

What is a child's diet, and who gives the food to the child when the mother goes out for work? [Probe]

What are the rationales behind delaying the early breastmilk introduction, discarding first milk from breasts? [Probe]

What are the worries of novice mothers and how far is social support essential for early breastmilk initiation soon after delivery? [Probe]

How important is household income, husband’s employment, peace at home, and time for the care of a baby? [Probe]

Does gender status also impact feeding? [Probe]

What are other things that we could not ask and that are important to tell us?

**Final Note: Thank you very much for your participation, and cooperation, and answers to questions. Please add anything that was not asked about?**
